# Exploring Numerical Correlations: Models and Thermodynamic Kappa

**DOI:** 10.3390/e27060646

**Published:** 2025-06-17

**Authors:** Nicholas V. Sarlis, David J. McComas, George Livadiotis

**Affiliations:** 1 Department of Astrophysical Sciences, Princeton University, Princeton, NJ 08544, USA; ns7888@princeton.edu (N.V.S.); dmccomas@princeton.edu (D.J.M.); 2 Physics Department, National and Kapodistrian University of Athens, Panepistimiopolis, 15784 Athens, Greece

**Keywords:** space plasmas, solar wind, heliosphere, kappa distributions, correlations, numerical experiment

## Abstract

McComas et al. (2025) introduced a numerical experiment, where ordinary uncorrelated collisions between collision pairs are followed by other, controlled (correlated) collisions, shedding light on the emergence of kappa distributions through particle correlations in space plasmas. We extend this experiment by introducing correlations indicating that (i) when long-range correlations are interwoven with collision pairs, the resulting thermodynamic kappa are described as that corresponding to an ‘interatomic’ potential interaction among particles; (ii) searching for a closer description of heliospheric plasmas, we found that pairwise short-range correlations are sufficient to lead to appropriate values of thermodynamic kappa, especially when forming correlated clusters; (iii) multi-particle correlations do not lead to physical stationary states; finally, (iv) an optimal model arises when combining all previous findings. In an excellent match with space plasmas observations, the thermodynamic kappa that describes the stationary state at which the system is stabilized behaves as follows: (a) When correlations are turned off, kappa is turning toward infinity, indicating the state of classical thermal equilibrium (Maxwell-Boltzmann distribution), (b) When collisions are turned off, kappa is turning toward the anti-equilibrium state, the furthest state from the classical thermal equilibrium (−5 power-law phase-space distribution), and (c) the finite kappa values are generally determined by the competing factor of collisions and correlations.

## 1. Introduction

Kappa distributions [1,2] that exhibit heavy tails are routinely used to describe space plasmas, as they can have both the core and tail features of the observed populations. Starting from the 1960s [3,4,5] the applications of kappa distributions now extend from the solar wind to planetary magnetospheres, out through the heliosheath beyond the termination shock, and even beyond the heliosphere to interstellar and astrophysical plasmas, see, e.g., [6,7,8,9,10,11,12,13,14,15,16,17] see also Table 1 of McComas et al. [18].

Kappa distributions are physically well founded and closely connected to nonextensive statistical mechanics [1,2,19,20,21,22,23,24,25,26,27], plasma physics [2,28,29,30,31,32], and thermodynamics [1,2,25,27,31,33,34,35,36,37,38,39,40]. They describe systems residing in stationary states that exist outside of classical thermal equilibrium [22,27,41]. Moreover, Livadiotis [37] and Livadiotis and McComas [26,35,42] showed that they are the most generalized particle energy distribution functions that are consistent with thermodynamics.

The *d*-dimensional kappa velocity u distribution is given by

(1) $$\begin{array}{c} P_{u}\left(u;\kappa_{0}\right)∼{\left[1+\frac{1}{\kappa_{0}}\frac{{\left(u-u_{b}\right)}^{2}}{\theta^{2}}\right]}^{-\kappa_{0}-1-\frac{1}{2}d}, \end{array}$$

(2) $$\begin{array}{c} P_{u}\left(u;\kappa_{0}\to \infty \right)∼\exp\left[-\frac{{\left(u-u_{b}\right)}^{2}}{\theta^{2}}\right] \end{array}$$

for a bulk velocity $u_{b}$, thermal speed θ (temperature *T* in speed dimensions) and kappa, $\kappa_{0}$, which parameterize the distribution. The thermodynamic kappa depends on the degrees of freedom *d* in a simple way that $\kappa \left(d\right)=\mathrm{const}.+\frac{1}{2}d$, where the constant constitutes the invariant kappa $\kappa_{0}$, which is independent of the degrees of freedom [36,43,44,45]. (For more on the notion and context of temperature and kappa, see refs. [2,11,26,30,31,36,37,43,45,46,47,48,49,50,51].) The limiting case of a Maxwell-Boltzmann (MB) distribution is obtained for $\kappa_{0}\to \infty$ (or κ→∞).

In terms of kinetic energy, $\epsilon =\frac{1}{2}m{\left(u-u_{b}\right)}^{2}$, to which we now turn the distribution in Equation (1) becomes:

(3) $$\begin{array}{c} P_{E}\left(\epsilon ;\kappa_{0}\right)∼{\left(1+\frac{1}{\kappa_{0}}\frac{\epsilon }{k_{B}T}\right)}^{-\kappa_{0}-1-\frac{1}{2}d}, \end{array}$$

(4) $$\begin{array}{c} P_{E}\left(\epsilon ;\kappa_{0}\to \infty \right)∼\exp\left(-\frac{\epsilon }{k_{B}T}\right). \end{array}$$

The kappa distribution is parameterized by the two independent thermodynamic quantities, the temperature and kappa. The latter is now understood to be a truly physically meaningful quantity that is as fundamental to thermodynamics as the temperature and this is the reason we now commonly refer to it as the thermodynamic kappa [31,36,37,38].

The physical understanding of kappa distributions has been finally established through the non-simple additivity of entropies due to the presence of the entropy defect [26,35,37,38,39,52,53]; see also [33,34,40]. The entropy defect is analogous to the mass defect, which quantifies the missing mass associated with the assembling of subatomic particles, but instead quantifies the missing entropy associated with the assembly of particle systems whose components have long-range correlations. Using the entropy defect, we are able to find the exact entropy of a system of particles with correlations while their energies, are described by a kappa distribution [35,37,45]. Indeed, the presence of correlations among the particle energies leads to a distribution of energies, that once stabilized, is uniquely described by kappa distributions [18]. In addition to this fundamental and macroscopic physical understanding from thermodynamics, various microphysical mechanisms have been shown to generate kappa distributions in space plasmas and other such systems. Some examples are superstatistics (i.e., the temperature is not fixed but has a special distribution, e.g., [40,54,55,56,57,58,59,60]), shock waves (e.g., [61]), turbulence (e.g., [28,62,63,64,65]), “pump mechanism” acceleration (e.g., [66]), Coulomb interactions [67,68,69], colloidal particles [70], and polytropes (e.g., [71,72,73,74,75]). What all of these mechanisms have in common is that they include long-range interactions that generate correlations among the particles and their energies.

**Figure 1:**
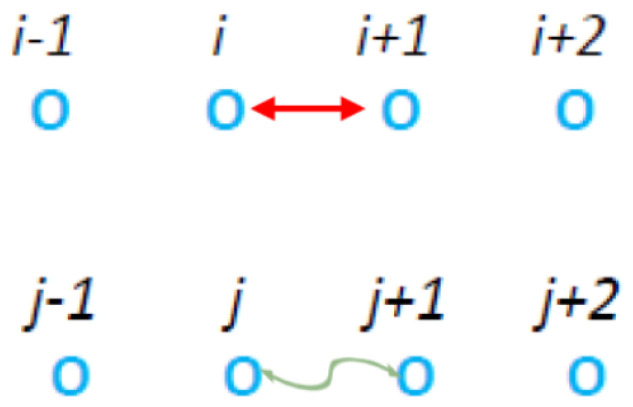
The original model suggested by McComas et al. [18]. In the first step a collision takes place between the *i* and the i+1 particle as shown by the double headed red arrow, while at the second time step “pseudo-collision”, or controlled collision, occurs between the randomly selected particle *j* and its adjacent particle j+1 (shown by the curly green line with arrowheads). McComas et al. [18] also allowed the repetition of the controlled collision step M−1(>0) times where each time we randomly select the position of the *j* particle. The model is complimented by periodic boundary conditions that means the particle next to the $i=i_{max}=N$ particle is the one at i=1.

A question arises from all of this new knowledge: Is there yet another, independent way to test our understanding of kappa distributions and their origin in correlations arising from long range interactions like interparticle electromagnetic interactions in plasmas? McComas et al. [18] provided a numerical experiment to test if a particle energy distribution function, which is described by a MB distribution in the absence of correlations, indeed generates a kappa distribution when correlations are introduced. In that study, we introduced a very simple model (Figure 1) that complements the above observational and theoretical work. This model is further discussed in Section 2.1.

The purpose of the present work is to further investigate the implications of this numerical model by introducing correlations in a broad variety of modeled ways, characterized by (i) long-range or short-range patterns, (ii) collision pairs, (iii) multi-particle interactions, and (iv) correlation clusters. The new models introduced and some of their results are presented in the various subsections of Section 2 while these findings are discussed in Section 3. Our conclusions are summarized in Section 4.

## 2. Numerical Models and Results

### 2.1. The Original Model

**Figure 2:**
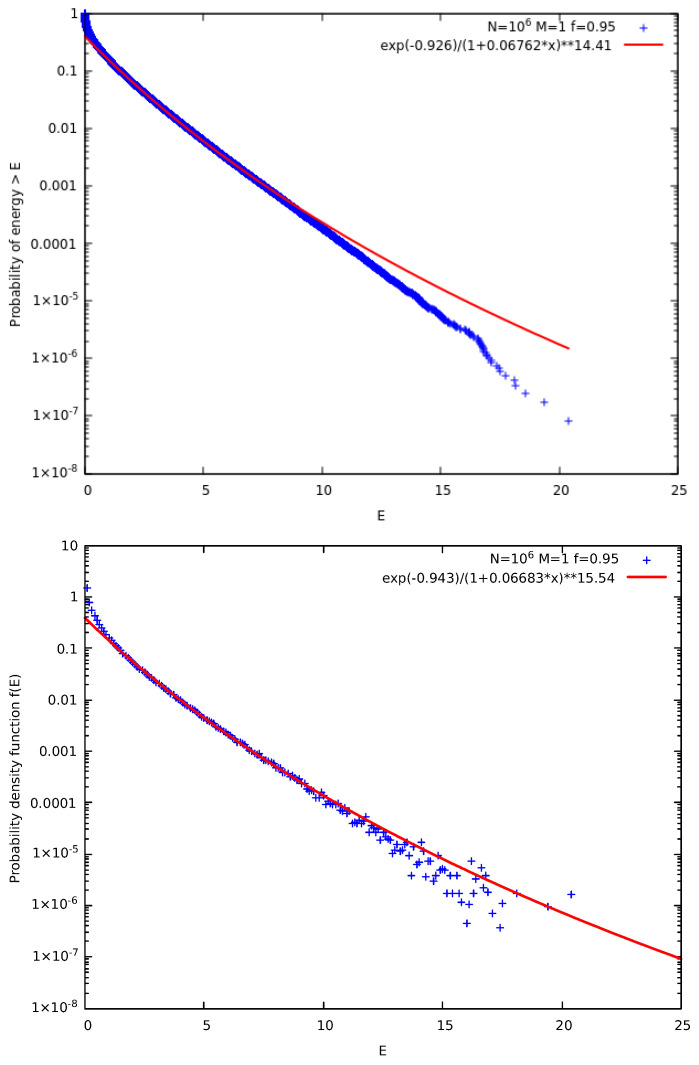
Results obtained from the original model of McComas et al. [18] for the case f=0.95 and M=1. Here, $N=10^{6}$ particles are used for $\tau =3\times 10^{5}$ MCS. The results of 12 replicas obtained through different initial conditions, due to different random number generator seeds, have been merged to improve the statistics. In the upper panel, we show the complementary cumulative distribution function $F_{ccd}\left(E\right)$, as in [18], while in the lower panel the pdf f(E) is plotted; in the figure key the symbol “∗∗” stands for exponentiation. The value of $\kappa_{0}$ obtained through Equation (20) is $\kappa_{0}=13.5\pm 0.3$.

McComas et al. [18] suggested a kinetic model in which each collision step that involves the collision pair (CP) of particles *i* and i+1 is followed by a correlation step (pseudo-collision or controlled collision), see Figure 1, in which we randomly select a “particle” say with index *j* and give a fraction *f* of its energy to the particle with index j+1. For symmetry reasons, with probability $\frac{1}{2}$ it is the particle with index j+1 that gives a fraction *f* of it to the *j* particle. There is also a possibility to repeat the correlation many times (*M* in total) with different random selections of index *j*. Upper panel of Figure 2 shows the results obtained for the complementary cumulative distribution function $F_{ccd}\left(E\right)$ that provides the probability of a particle to have an energy larger than *E*. It is noteworthy that $F_{ccd}\left(E\right)$ is a direct outcome of the simulation (see Appendix A in [18]) since it can be obtained without any additional calculation apart from sorting the simulation calculated particle energies. When considering *N* particles in such simulations, we usually make $\tau =3\times 10^{5}$ Monte Carlo Steps (MCS) with each MCS corresponding to randomly selecting *N* CPs and performing the M correlation steps. To obtain Figure 2 we used $N=10^{6}$ particles initially distributed in a MB distribution with average energy 0.5 and performed $\tau \times N=3\times 10^{11}$ times the process depicted in Figure 1. In the lower panel of Figure 2, we depict the corresponding probability density function (pdf) f(E) which was calculated by kernel estimation, see, e.g., Refs. [76,77]. In order to understand the functional forms of $F_{ccd}\left(E\right)$ and f(E) depicted by continuous lines in Figure 2, we now recall some properties of kappa distributions [35,37].

Assuming that our simple model involves two-dimensional collisions in a system with *N* particles. We retrieve the values of the speeds (magnitudes of the two-dimensional velocities) after the collision and produce their complementary cumulative distribution (ccd) function. Once the distribution resides in a stationary state, it can be modeled by a kappa distribution.

The *d*-dimensional kappa distribution velocity distribution is given by Equation (1), which in the case of zero flow speed becomes

(5) $$P_{u}\left(u;\kappa_{0}\right)∼{\left[1+\frac{1}{\kappa_{0}}\frac{u^{2}}{\theta^{2}}\right]}^{-\kappa_{0}-1-\frac{1}{2}d}$$

or, in terms of the kinetic energy $P_{E}\left(\epsilon \right)=P_{u}\left(u=\sqrt{\frac{2}{m}\epsilon }\right)$, Equation (3). The density of states for integrating the distribution of Equation (3) is $g\left(\epsilon \right)∼\epsilon^{\frac{1}{2}d-1}$. Therefore, the normalization of this distribution is given by

(6) $$1=\int_{0}^{\infty }P_{E}\left(\epsilon \right)g\left(\epsilon \right)d\epsilon ,$$

or, the corresponding normalization constant is

(7) $$A=\int_{0}^{\infty }{\left(1+\frac{1}{\kappa_{0}}\frac{\epsilon }{k_{B}T}\right)}^{-\kappa_{0}-1-\frac{1}{2}d}\epsilon^{\frac{1}{2}d-1}d\epsilon .$$

This integration defines the kappa adaptation of the Gamma function (see: Appendix A of Livadiotis and McComas [22]). Moreover, the corresponding energy ccd function is given by the kappa adaptation of the incomplete gamma function, i.e.,

(8) $$\Gamma_{\kappa }\left(x,d\right)∼\int_{x}^{\infty }P_{E}\left(\tilde{x}\right)g\left(\tilde{x}\right)d\tilde{x}∼\int_{x}^{\infty }{\left(1+\frac{1}{\kappa_{0}}\tilde{x}\right)}^{-\kappa_{0}-1-\frac{1}{2}d}{\tilde{x}}^{\frac{1}{2}d-1}d\tilde{x}.$$

In the two-dimensional case, this has a simple analytical form, i.e.,

(9) $$\Gamma_{\kappa }\left(x,2\right)∼\int_{x}^{\infty }P_{E}\left(\tilde{x}\right)g\left(\tilde{x}\right)d\tilde{x}∼\int_{x}^{\infty }{\left(1+\frac{1}{\kappa_{0}}\tilde{x}\right)}^{-\kappa_{0}-2}d\tilde{x},$$

or

(10) $$\Gamma_{\kappa }\left(\epsilon ,2\right)=\frac{\int_{\epsilon }^{\infty }{\left(1+\frac{1}{\kappa_{0}}\frac{\tilde{\epsilon }}{k_{B}T}\right)}^{-\kappa_{0}-2}d\tilde{\epsilon }}{\int_{0}^{\infty }{\left(1+\frac{1}{\kappa_{0}}\frac{\tilde{\epsilon }}{k_{B}T}\right)}^{-\kappa_{0}-2}d\tilde{\epsilon }},$$

which gives

(11) $$\Gamma_{\kappa }\left(\epsilon ,2\right)={\left(1+\frac{1}{\kappa_{0}}\frac{\tilde{\epsilon }}{k_{B}T}\right)}^{-\kappa_{0}-1},$$

where this modeled ccd function must be multiplied by the involved number of particles before fitting.

First, we may fit the no-correlation case that leads to MB distribution, in order to find the temperature *T*,

(12) $$F_{ccd\infty }=\exp\left(-\frac{\epsilon }{k_{B}T}\right),$$

where the ccd refers to one particle energy distribution (it has been normalized to the number of particles).

Next, we proceed to the correlation case, where we expect to have the same parameter value $k_{B}T$. (Nevertheless, the way of inducing the correlations may affect this parameter, namely, a large number of “particles” may have zero speed). Therefore, we choose to fit again the ccd function with two fit parameters, i.e.,

(13) $$F_{ccd\kappa }\left(\epsilon \right)={\left(1+\frac{1}{\kappa_{0}}\frac{\epsilon }{k_{B}T}\right)}^{-\kappa_{0}-1}.$$

In addition, the two-dimensionality assumption may be suffering from fluctuations of the degrees of freedom, which is called and determined by the phenomenological name of “effective dimensionality”; here, we also investigate this generalized case of effective dimensionality,

(14) $$\Gamma_{\kappa }\left(\epsilon ,2\right)=\frac{\int_{\epsilon }^{\infty }{\left(1+\frac{1}{\kappa_{0}}\frac{\tilde{\epsilon }}{k_{B}T}\right)}^{-\kappa_{0}-1-\frac{1}{2}d}d\tilde{\epsilon }}{\int_{0}^{\infty }{\left(1+\frac{1}{\kappa_{0}}\frac{\tilde{\epsilon }}{k_{B}T}\right)}^{-\kappa_{0}-1-\frac{1}{2}d}d\tilde{\epsilon }},$$

leading to the ccd function

(15) $$F_{ccd\kappa }\left(\epsilon \right)=\frac{A\left(\frac{\epsilon }{\kappa_{0}k_{B}T}\right)}{A(0)},$$

where

(16) $$A\left(x\right)\equiv \int_{x}^{\infty }{\left(1+\xi \right)}^{-\kappa_{0}-1-\frac{1}{2}d}\xi^{\frac{1}{2}d-1}d\xi .$$

This integral can be estimated in terms of the Gauss hypergeometric function x2F1(α,β;γ;z), see Equation (3.194.2) of Gradsteyn and Ryzhik [78], i.e.,

(17) A(x)=x2F1(κ0+1+12d,κ0+1;κ0+2;−1/x)(κ0+1)xκ0+1,

while

(18) $$A\left(0\right)=B\left(\frac{1}{2}d,\kappa_{0}+1\right),$$

due to Equation (3.194.3) of [78] and B(x,y) is the beta function.

In this paper, we exclusively use Equation (13), while its generalizetion, Equation (15), can be used for further future analyses. Additionally, we use the pdf f(ϵ) obtained from Equation (13)

(19) $$f\left(\epsilon \right)=-\frac{dF_{ccd\kappa }\left(\epsilon \right)}{d\epsilon }∼{\left(1+\frac{1}{\kappa_{0}}\frac{\epsilon }{k_{B}T}\right)}^{-\kappa_{0}-2}.$$

In order to estimate the thermodynamic kappa from the simulation results, we adopt Equations (13) and (19) to simultaneously fit the ccd and the pdf of the particle energy distribution, respectively, using [79] the nonlinear least-squares Marquardt-Levenberg algorithm. A functional form of g(E)=n−klog(1+a·E) is used for fitting the logarithms of ccd or pdf, as shown in Figure 2, and we select the fitting energy range so that the $k_{pdf}-k_{ccd}\approx 1$, since $k_{pdf}$ and $k_{ccd}$ correspond to $\kappa_{0}+2$ and $\kappa_{0}+1$, respectively. Using these, we obtain

(20) $$\kappa_{0}=\frac{k_{pdf}+k_{ccd}-3}{2}.$$

### 2.2. Long-Range Models

#### 2.2.1. The Short-Range Case Close to the CP

**Figure 3:**
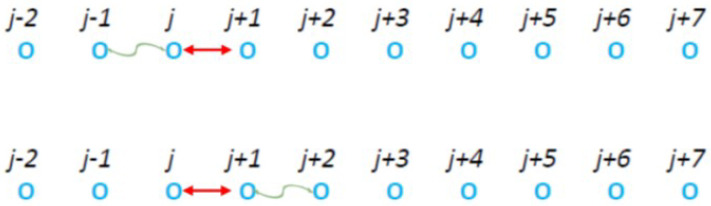
Two equivalent models in which during the second time step the “pseudo-collision”, or controlled collision, occurs between one particle of the CP (linked with the red double headed arrow) that collided during the first time step and its nearest neighbor (shown by the curly green line with arrowheads).

In order to study the influence of a long-range interaction in the correlation step of Figure 1, we firstly investigated the case of the short-range nearest neighbor interactions exactly adjacent to the CP as shown in Figure 3.

**Figure 4:**
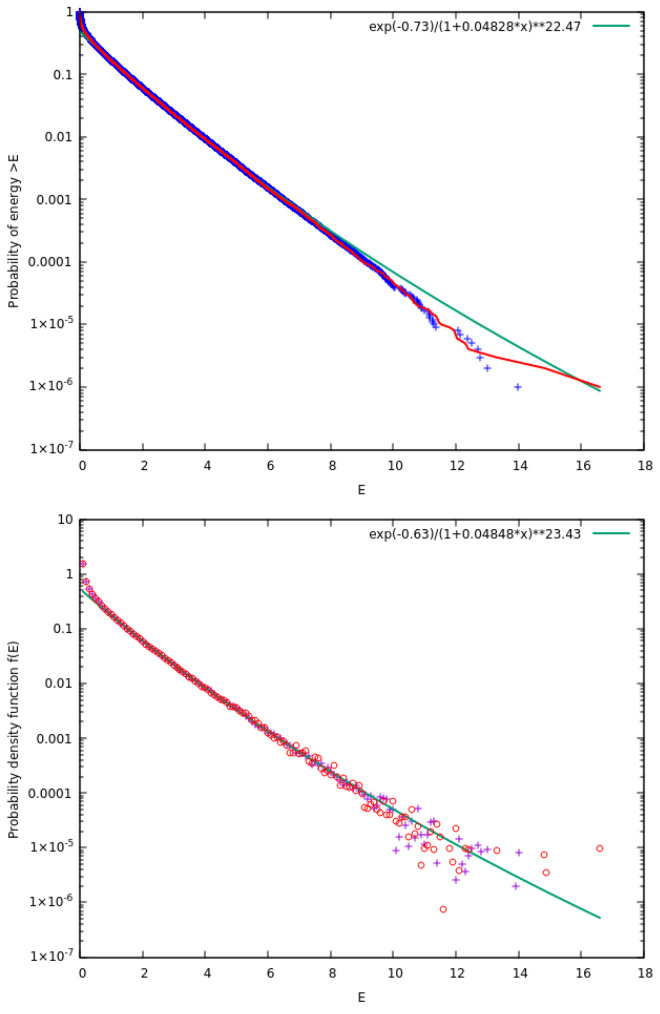
Results obtained from the two models of Figure 3 for the case f=0.95. Here, $N=10^{6}$ particles with $\tau =3\times 10^{5}$ MCS. In the upper panel, we show the complementary cumulative distribution function $F_{ccd}\left(E\right)$ with blue plus symbols for the model in upper panel of Figure 3 and red continuous line for the one in the lower panel of Figure 3; in the figure key the symbol “∗∗” stands for exponentiation. The lower panel depicts the two pdfs f(E) for the model in the upper and lower panel of Figure 3 with plus symbols and open circles, respectively. The green lines correspond to the simultaneous fit by kappa distributions of Equations (13) and (19) and leads through Equation (20) to a value of $\kappa_{0}=21.5\pm 0.3$.

Simulations of the two models shown in Figure 3 lead to the results presented in Figure 4. As expected the two models lead to identical results with a value of $\kappa_{0}=21.5\pm 0.3$. We note that this value of $\kappa_{0}$ is significantly larger than that obtained in Figure 2 for the original model suggested by McComas et al. [18].

#### 2.2.2. The Long-Range Case Around the CP

**Figure 5:**
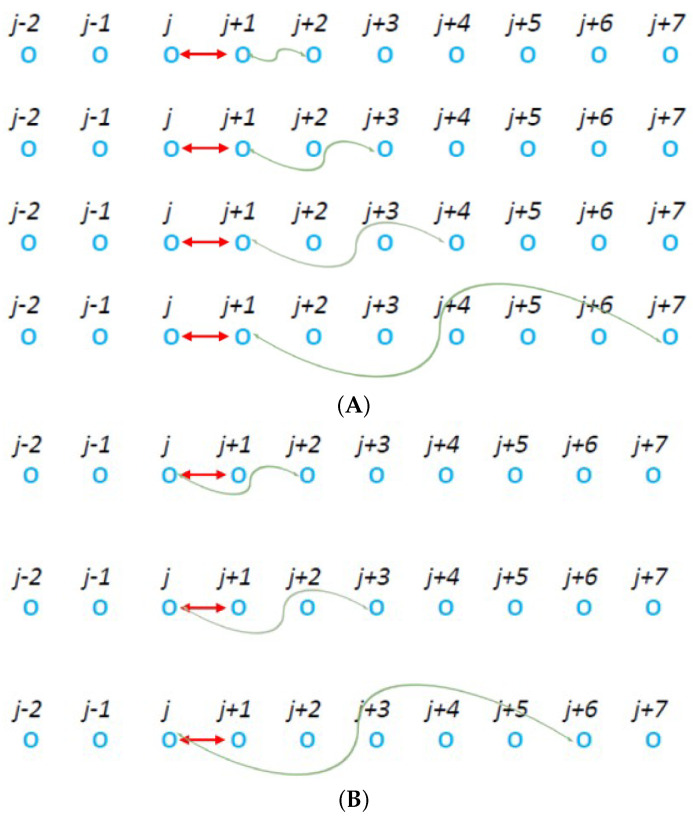
The two models of varying long-range correlations at a distance *R* that have been studied in Section 2.2.2. From top to bottom and for the class of the models shown in panel (**A**) R=1, 2, 3, and 6 while for those shown in (**B**) we set R=1, 2, and 5. In all cases, the CP is at the particles *j* and j+1 and is indicated by the red double headed arrow.

We next proceed to the introduction of (long) range interactions mediated at a distance *R* through the correlation step. Specifically, we considered two classes of models: Model “a” which is shown in Figure 5A and model “b” shown in the lower panel of the same figure, i.e., Figure 5B. In model “a”, the correlation step takes place between one particle of the CP and its nearest, second nearest (R= 2), third nearest (R= 3) neighbor, etc., as shown in Figure 5A. Model “b” allows the correlation step to occur between one particle of the CP and the nearest (R= 1), second nearest (R= 2), third nearest (R= 3), etc., of the other particle of the CP, see Figure 5B.

**Figure 6:**
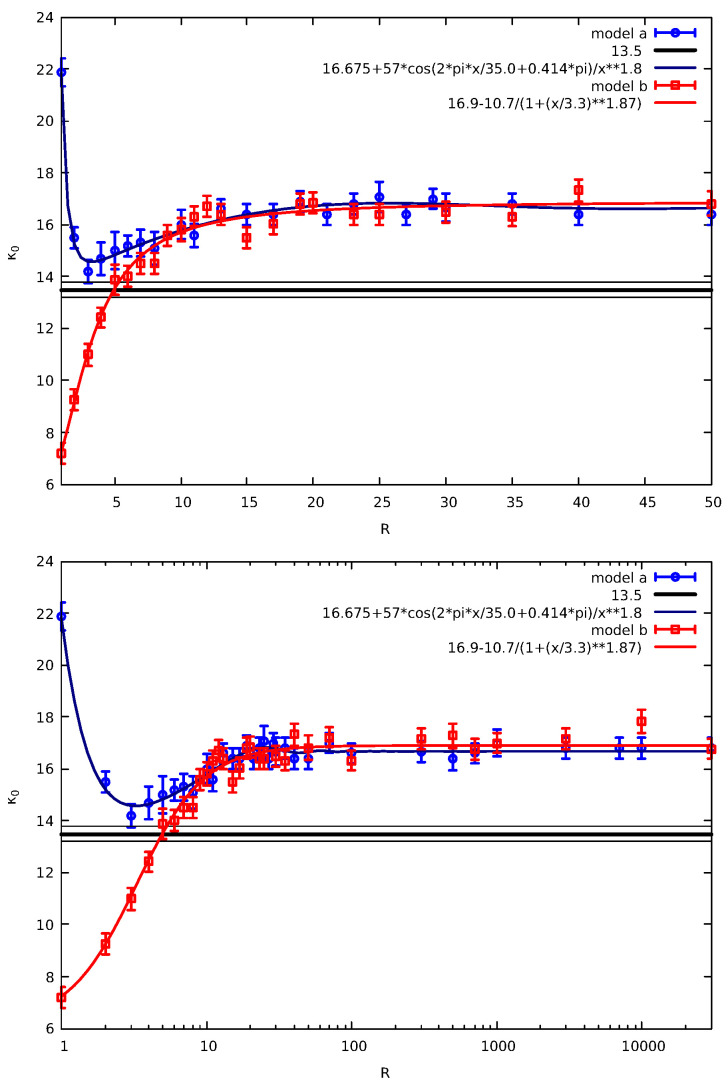
Results of models“a” (blue) and “b” (red) for $\kappa_{0}$ vs. the range *R* used in the correlation step (see Figure 5), plotted on linear-linear and linear-log scales; in the figure key the symbol “∗∗” stands for exponentiation. The continuous lines in each case have been drawn as a guide to the eye. The thick horizontal black line indicates the value of $\kappa_{0}$ estimated in Figure 2 while the thinner black lines the corresponding error.

For these cases, we considered f=0.95 and fit $F_{ccd}\left(E\right)$ in a given energy range (E∈[1.2,12]) for the distribution of energies obtained by 10 replicas of $N=10^{6}$ particles. We selected this method because it allows fast calculation of $\kappa_{0}$ as well as a direct comparison of $\kappa_{0}$ between different values of the distance *R*. Thus, we obtained the results shown in Figure 6. The upper panel of Figure 6 depicts the results in a linear plot while the lower panel is on a semi-logarithmic scale.

Figure 6 reveals that models “a” and “b” result in $\kappa_{0}$ values that resemble inter-atomic potentials. One with repulsive part for small R in “a” and a fully attractive one in model “b”. We note that the ability of thermodynamic kappa to comprise interaction potentials was first discussed by Livadiotis [80]. The fact that model “b” exhibits stronger correlations is related to the fact that in such a model particle j+1 of the CP resides (see Figure 5B) between the correlated particles at positions *j* and j+1+R. This adds correlations in the model as can be understood through the following example: Consider the model of the first row of Figure 5A and suppose that after selecting *j* and j+1 as CP at a later selection the CP is that of the particles j+1 and j+2. Upon performing this later step, the correlation between particles j+1 and j+2 that was previously induced is canceled due to the ordinary collision between j+1 and j+2 particles. Such a cancellation does not occur, however, if we consider the model of the first row of Figure 5B. We note that the continuous lines shown in Figure 6 include the range *R* exponentiated to ≈1.84 which is close to two, which corresponds to an electric dipole potential [81]. We also observe that the minimum $\kappa_{0}$ for model “a” is at R≈3.5 while the range scale appearing as denominator in the fitting curve of model “b” is 3.3.

To understand a possible geometric origin of these two numbers, we recall the percolation problem in 1-dimension, (see, e.g., [82] and references therein). As said, during a MCS we select as many collision steps as the number of particles *N*. Thus, each particle has a mean value of one to be selected as the *j* particle in Figure 5 and the number of times a particle has been chosen in a MCS follows Poisson distribution with mean value λ=1. Now, the probability a given particle that is not selected as the *j* particle during a MCS is given by $p_{0}=\exp\left(-\lambda \right)=\exp\left(-1\right)\approx 0.368$. We now turn to the 1-dimensional site percolation problem in which we have a probability $p(=1-p_{0})$ a site to be occupied (visited at least once in our problem). In percolation, we are interested in finding the size of clusters of contiguous occupied sites. In the 1-dimensional site percolation problem, the cluster number density is given by (see Equation (2.20) in [82]) $n\left(s,p\right)=\left(1-p^{2}\right)\exp\left(-s/s_{\xi }\right)$, where the characteristic scale is $s_{\xi }=-1/\ln p$ which leads to $s_{\xi }\approx 2.18$ in our case. If we now consider that the average cluster size 〈s〉 is given by Equation (2.26) of [82], i.e., 〈s〉=(1+p)/(1−p), we find that 〈s〉≈4.435. We observe that the average $(s_{\xi }+\langle s\rangle )/2\approx 3.3$ of these two characteristic numbers of the 1-dimensional percolation problem compares favorably with the scale of around 3.4 identified by our models “a” and “b”.

Interestingly, the lower panel of Figure 6 shows that both models lead to the same value of $\kappa_{0}$ for very large *R* which is higher than that obtained for the original model in Figure 2. This is consistent with the view that the “pseudo-collision” of one particle of the CP leads to smaller correlations compared to those obtained when two neighbors at a random (possibly very distant from the CP) site “pseudo-collide”.

#### 2.2.3. Long-Range Correlations at Range *m*

**Figure 7:**
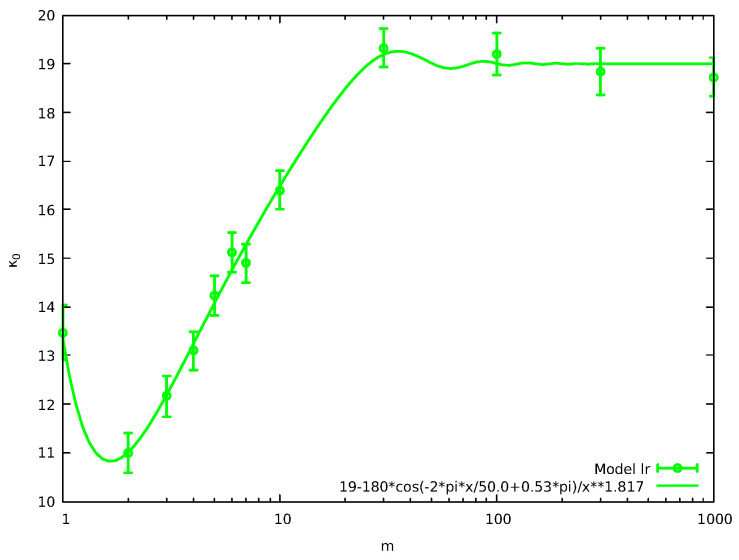
Results of the “lr” model discussed in Section 2.2.3: $\kappa_{0}$ vs the range *m* as estimated by fitting $F_{ccd}\left(E\right)$ in the energy range E∈[1.5,15] to the distribution of energies obtained by at least 10 replicas of $N=10^{6}$ particles in each case. The continuous green line has been drawn as a guide to the eye. The $\kappa_{0}$ value at m=1 corresponds that estimated in Figure 2.

Finally, in order to investigate the case of long-range “pseudo-collisions”, we run variations of the standard model of Figure 1 in which after the ordinary collision between the particles of the CP, the “pseudo-collision” takes place between the particles *j* and j+m. Thus, *m* measures the range of these long-range “pseudo-collisions”, we name the resulting model as model “lr” and it influences $\kappa_{0}$ as shown in Figure 7.

Inspection of Figure 7 reveals two common features with those of Figure 6: The power law exponent in the green fitting line is again close to two and $\kappa_{0}$ stabilizes for large values of *m*. The limiting value, however, is now $\kappa_{0}=19$ somewhat larger than ≈16.8 that was in Figure 6. This increase of $\kappa_{0}$ shows that in current model “lr” the correlations at large *m* are smaller than those for large *R* at models “a” and “b”. This is reasonable since in the latter two models one of the particles of the CP takes part in the “pseudo-collision” reducing the randomization introduced by the ordinary collision in the system.

### 2.3. Multiparticle Interactions

**Figure 8:**
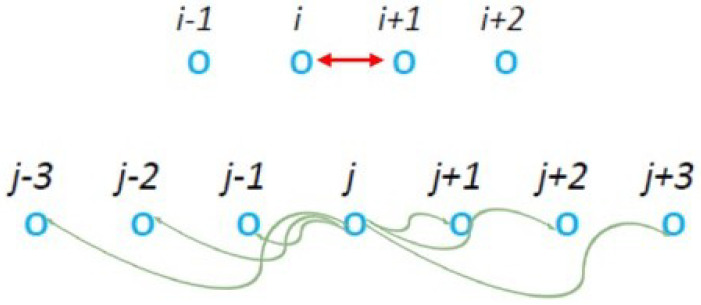
Class of models “m” in which the “donor” particle at *j* gives a part *f* of its energy to its six nearest neighbors j−3, j−2, j−1, j+1, j+2, j+3 with percentages $r_{-3}$, $r_{-2}$, $r_{-1}$, $r_{1}$, $r_{2}$, and $r_{3}$, respectively, after the collision step at the CP *i* and i+1.

In order to create a correlated cluster around the *j* particle, we next considered the models shown in Figure 8 in which, after the collision step at the CP of *i* and i+1 particles, a multiparticle “pseudo-collision” step occurs centered at a randomly chose particle at *j*. We call these models “m” from the multiparticle “pseudo-collision” that distributes simultaneously the energy of the “donor” particle at *j* to its neighbors at j+s with percentage $r_{s}$ for *s* = −3, −2, −1, 1, 2, and 3 with $\sum_{s=-3}^{3}r_{s}=1$, where $r_{0}=0$.

**Figure 9:**
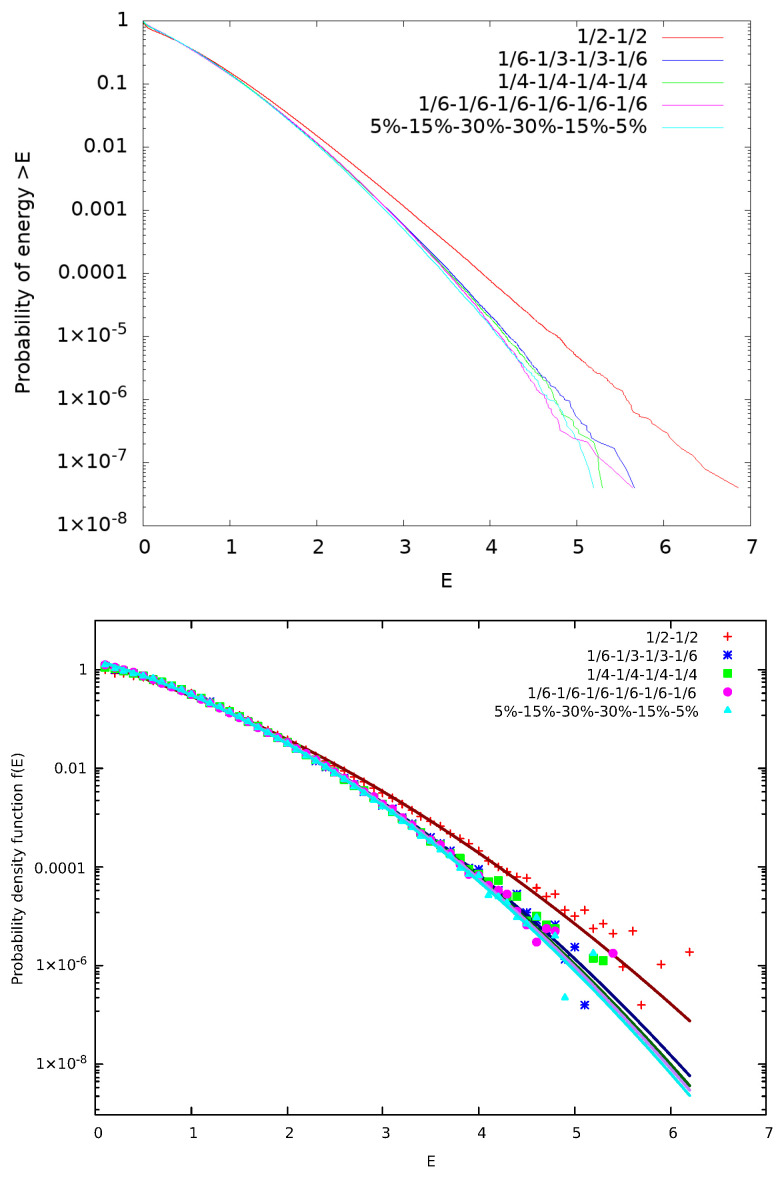
Results obtained from various models “m” with different distribution $r_{s}$ (shown in the legend) of the energy of the donor particle at *j* to its neighbors (see Figure 8) for the case f=0.95. Here, $N=10^{6}$ particles have been considered for $\tau =3\times 10^{5}$ MCS and the results of 24 replicas obtained through different initial conditions due to different random number generator seeds have been merged to improve the statistics. In the upper panel, we show the complementary cumulative distribution function $F_{ccd}\left(E\right)$, while in the lower panel the pdf f(E) is plotted. The continuous curves in the lower panel are parabolas showing that “m” models do not lead to kappa distributions but rather to Gaussians.

We simulated five such “m” models with various distributions $r_{s}$ of energy to the neighbors of particle *j*, but our results—shown in Figure 9—reveal that such multiparticle collision models do not lead to kappa distributions. They result in Gaussian like distributions represented by parabolas in the log-lin plots of the lower panel of Figure 9. This points to the fact that multiparticle “pseudo-collisions” are as unlikely as multiparticle ordinary collisions. Particles in nature collide in pairs!

### 2.4. Correlation Clusters

**Figure 10:**
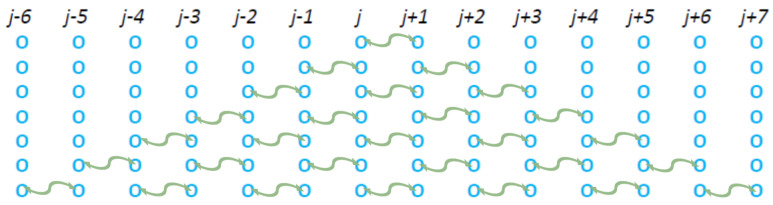
A class of models, labeled C1 to C7 according to the number of correlation time steps followed from top to bottom, where after the collision at the CP, we attempt to form a correlated cluster by simultaneously pseudo-colliding the particles around the *j* and j+1 pair. Each curly green line indicates a pseudo-collision and the number of steps increases by one each time we move down the rows that form the edges of the pyramid structure.

As we saw in the previous subsection, correlation clusters that can be described by kappa distributions cannot be formed by multiparticle collisions. Thus, we introduce now the notion of the correlation time step (see the multiple lines of the model depicted in Figure 10). A correlation time step is a time step during which we perform “simultaneously” pseudo-collision between adjacent pairs of particles in the neighborhood of the *j* and j+1 particles that had the initial pseudo-collision, see the top line of Figure 10. During a correlation time step, we pseudo-collide these particles in a way that the order performing the pseudo-collisions does not affect the result. This way the correlation steps evolve and form the pyramid like structure shown in Figure 10 having on top the pair of particles *j* and j+1. We call these models as “C#” where # is the number of correlations steps made. For example, model “C1” is equivalent to the original model suggested by McComas et al. [18] shown in Figure 1. As the correlation cluster grows so does the correlation between the particles giving rise to smaller and smaller $\kappa_{0}$.

**Figure 11:**
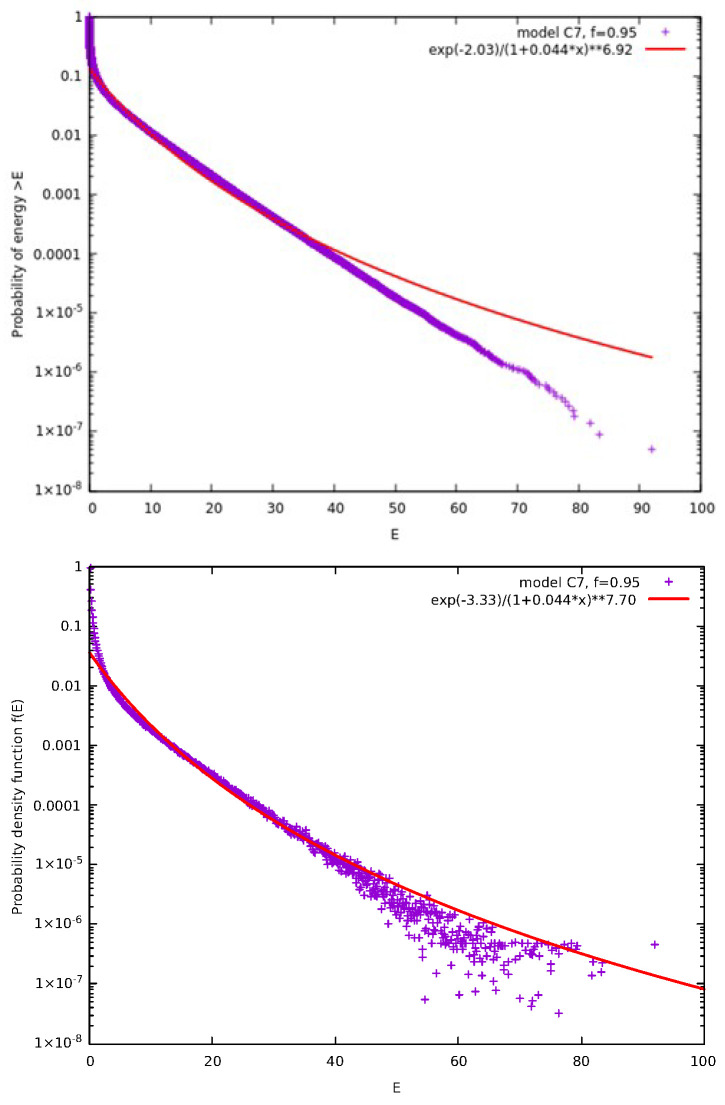
Results obtained from model C7, i.e., the whole model depicted in Figure 10 with seven correlation time steps for the case f=0.95. Here, $N=10^{6}$ particles have been considered for $\tau =3\times 10^{5}$ MCS and the results of 24 replicas obtained through different initial conditions due to different random number generator seeds have been merged to improve the statistics. In the upper panel, we show the complementary cumulative distribution function $F_{ccd}\left(E\right)$, while in the lower panel the pdf f(E) is plotted; in the figure key the symbol “∗∗” stands for exponentiation. The continuous curves in both panels show the simultaneous fits for both the ccd and the pdf with $\kappa_{0}=5.8\pm 0.3$.

Figure 11 shows the results obtained for the model C7 for f=0.95. We can see that the resulting $\kappa_{0}$ is smaller than all the $\kappa_{0}$ calculated so far for f=0.95 including those discussed in our previous paper [18] see, e.g., Figure 3 of McComas et al. [18].

#### Limiting the Correlation Cluster

**Figure 12:**
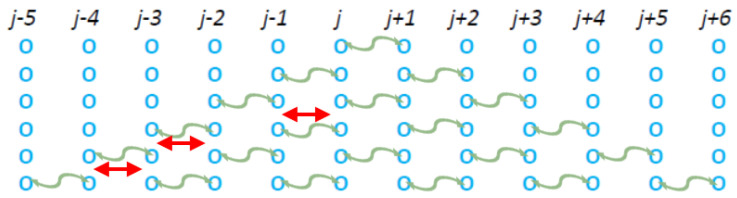
By adding ordinary collisions (denoted by the red double headed arrows) between the steps of the C# class models, we limit the effect of the correlation cluster. These models are labeled as C4-t1 for the model with 4 correlation time steps and one ordinary collision, C5-t2 for the model with 5 correlation time steps and two ordinary collisions, and C6-t3 for the model with 6 correlation time steps and 3 ordinary collisions that corresponds to the totality of the figure.

If our simple model was to describe real systems, there must be a mechanism to compensate for an ad infinitum increasing size of the correlation cluster. For this reason, we suggest the addition of ordinary collisions between the correlation time steps, see Figure 12.

In these models, labeled as “C(#)_1_-t(#)_2_” where (#)_1_ is the number of correlation time steps and (#)_2_ is the number of ordinary collisions occurring in between them, we assume that the ordinary collision can occur in between every pair of particles that was involved in the previous correlation time steps and we select one of these pairs at random as CP. One such ordinary collision is enough to limit the cluster and the rest of the tree-like structures emanating from this CP in the following correlation time steps, making them uncorrelated from the rest of the initial correlated cluster. Actually, they are equivalent to smaller (#)_1_ correlated clusters, so this is a way to effectively cancel correlation time steps.

**Figure 13:**
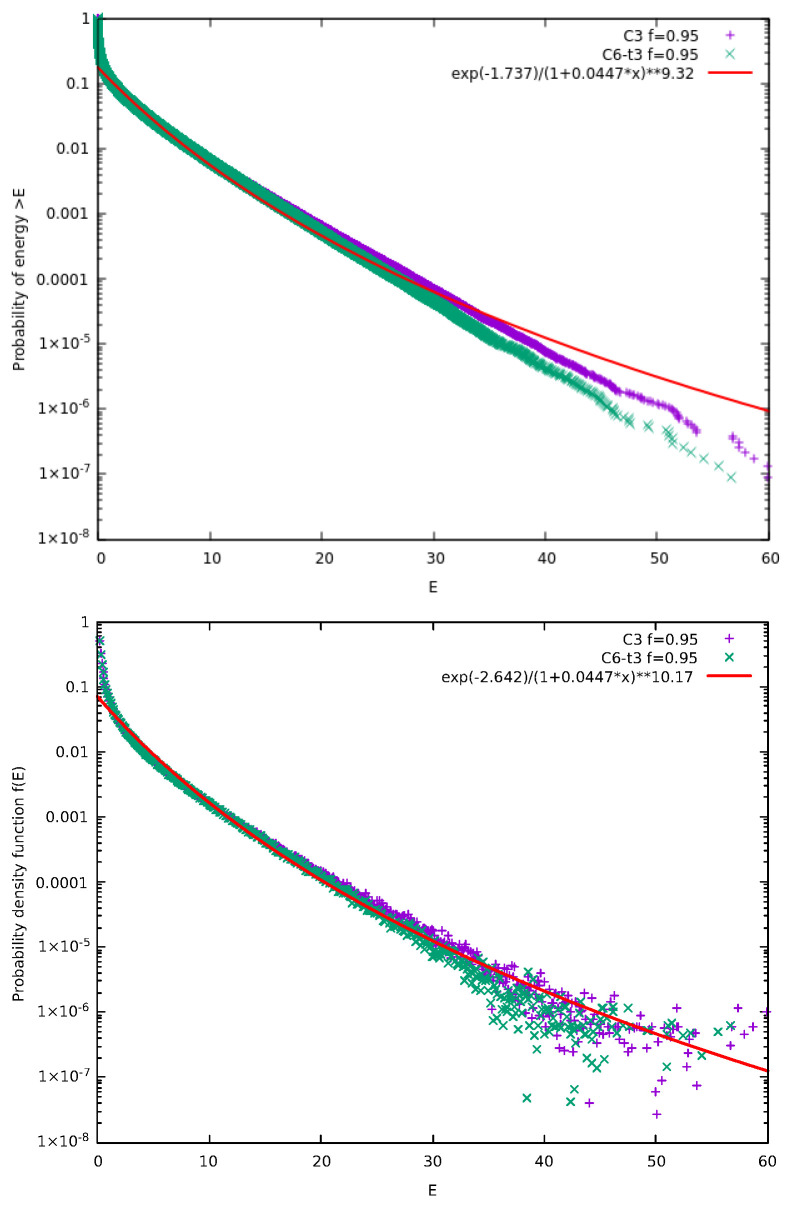
Results obtained from models C6-t3, i.e., the model depicted in Figure 12 for the case f=0.95, and C3. Here, $N=10^{6}$ particles have been considered for $\tau =3\times 10^{5}$ MCS and the results of 24 replicas obtained through different initial conditions due to different random number generator seeds have been merged to improve the statistics. As mentioned in the text, we also include the results obtained from the model C3 of Figure 10, in which we consider only three correlation time steps, that give rise to curves which are higher than those of the C6-t3 model pointing to stronger correlation and smaller $\kappa_{0}$. In the upper panel, we show the complementary cumulative distribution function $F_{ccd}\left(E\right)$, while in the lower panel the pdf f(E) is plotted; in the figure key the symbol “∗∗” stands for exponentiation. The continuous curves in both panels show the simultaneous fits for both the ccd and the pdf of C6-t3 with $\kappa_{0}=8.2\pm 0.3$.

Figure 13 presents the results obtain from the from the C6-t3 model for f=0.95. In the same figure, we also present the results obtained by the C3 model. An inspection of Figure 13 reveals that the three ordinary collisions of the C6-t3 model are enough to compensate for the three more correlation time steps compared to the C3 model. Indeed, the resulting energy distributions reveal a higher correlation in C3 model than in C6-t3 since the C3 model curves lie above those for C6-t3 (cf. for the C3 model the corresponding $\kappa_{0}=6.9\pm 0.3$).

## 3. Discussion

McComas et al. [18] introduced the model of Figure 1 that consists of an ordinary collision shown in the first line of the figure and a pseudo-collision or controlled collision shown in its second line. We note that McComas et al. [18] allowed for a number M≥1 of pseudo-collisions per ordinary collision. In Figure 2 of [18] the case for M=2 with f=0.95 has been presented. Here, in comparison to that figure we show what happens with the correlations amongst the energies of adjacent particles.

**Figure 14:**
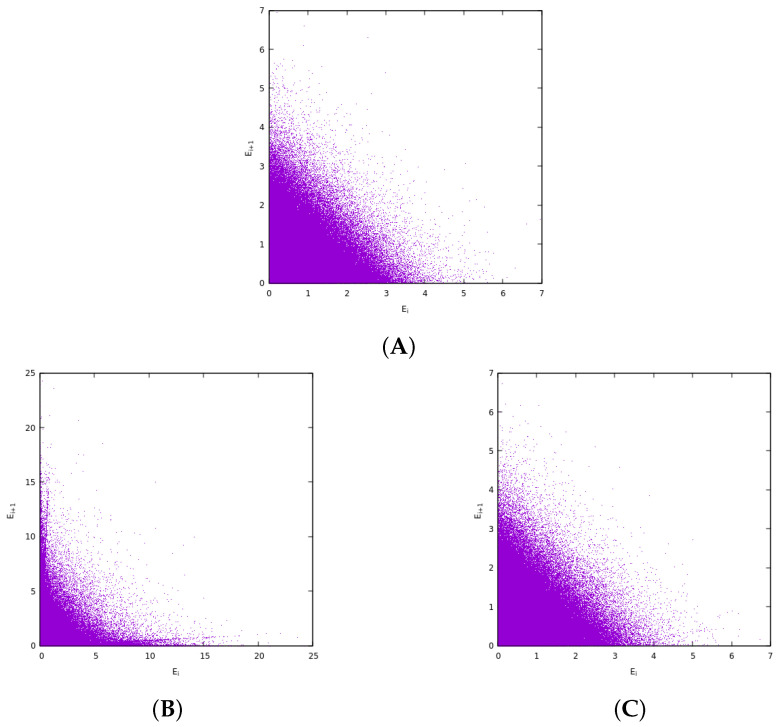
Results obtained for the energy distribution of adjacent particles *i* and i+1 when (**A**) only the collision step is involved in the calculation, i.e., the upper line of Figure 1, (**B**) after $\tau =3\times 10^{5}$ MCS for the model with M=2 and f=0.95 (results of which were presented in Figure 2 of McComas et al. [18]), and (**C**) after $\tau =3\times 10^{5}$ MCS with only collision steps but starting from the distribution shown in (**B**). Here, we show the distribution of $E_{i}$ and $E_{i+1}$ by simply depicting the points ($E_{i}$, $E_{i+1}$) as dots (forming a cloud) and $N=10^{6}$ particles.

**Figure 15:**
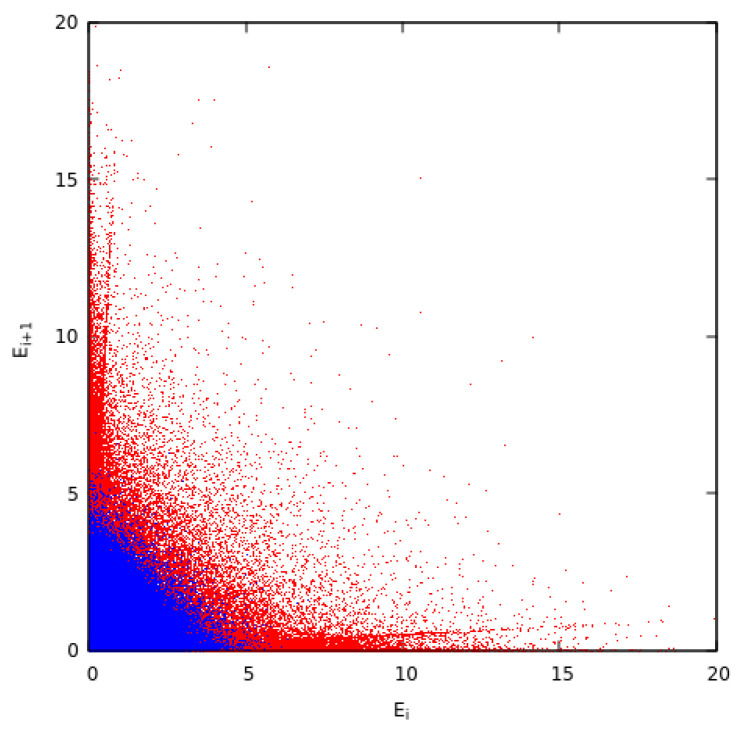
The same as Figure 14 but on an expanded scale where we depict the distribution of Figure 14A (blue dots) on top of the distribution of Figure 14B (red dots).

Figure 14A depicts $E_{i+1}$ versus $E_{i}$ for the case where we focus on the role of ordinary collisions, which result from all kinds of short-range interactions that leave the particles free after the collision. We observe that the energies are not correlated and no particular pattern can be identified. The exponentially distributed and independent energies $E_{i}$ and $E_{i+1}$ determine the observed, almost triangular shape. Note that this statistical behavior and plot can be also observed when plotting consecutive exponentially distributed random numbers obtained from a random number generator. On the other hand, when pseudo-collisions come into play in Figure 14B patterns emerge, see for example the two lines that appear close to the horizontal and the vertical axes as well as the significant modulation of the initial triangular shape to that of a hyperbolic boundary. These two latter structures are the characteristic ones that can be seen in Figure 15. Finally, in Figure 14C we depict the distribution after evolving from the state of Figure 14B without any controlled collisions and only carrying out ordinary collisions. We see that the distribution returns to that of uncorrelated energies, i.e., it is the same as in Figure 14A, which corresponds to the MB distribution.

**Figure 16:**
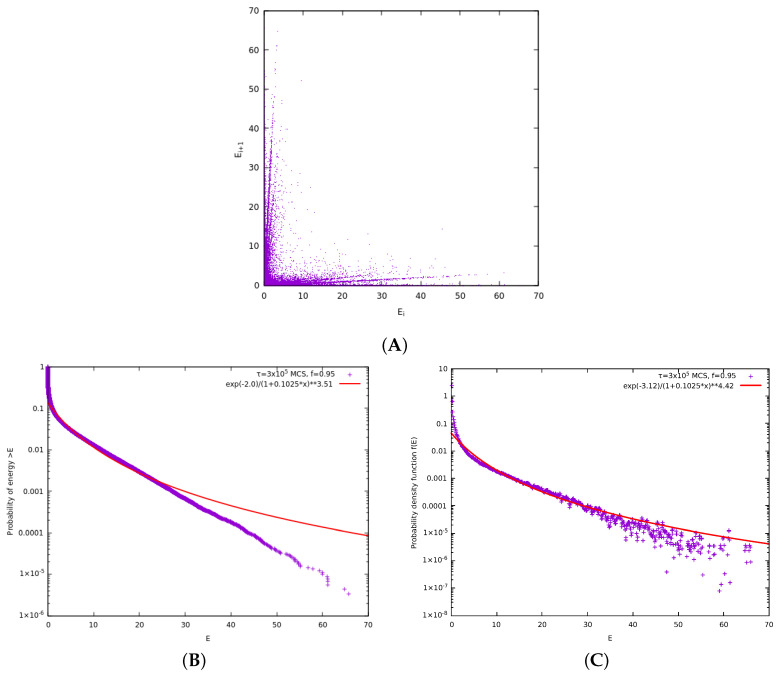
Here we consider $N=10^{6}$, f=0.95 and only controlled collisions take place starting from a MB distribution for the energies, see, e.g., Figure 14A. After $\tau =3\times 10^{5}$ MCS we have: (**A**) The energy distribution of adjacent particles *i* and i+1, (**B**) the complementary cumulative distribution function $F_{ccd}\left(E\right)$, and (**C**) the pdf f(E). The continuous curves in the lower two panels show the simultaneous fits for both the ccd and the pdf with $\kappa_{0}=2.5\pm 0.3$.

Next we explore what happens if we remove ordinary collisions and keep only the pseudo-collision depicted in the second line of Figure 1. As initial conditions, we assume that the energies are MB distributed, i.e., they follow an exponential distribution. The results obtained for such a simple controlled collision model are shown in Figure 16. An inspection of Figure 16A and its comparison with Figure 14 reveals that the elimination of ordinary collisions leads to stronger correlations which are shown with more elaborate patterns in Figure 16A than those in Figure 14B. This is also reflected in the kappa distributions that fit the ccd and the pdf of the energy. The latter two curves are shown in Figure 16B and Figure 16C, respectively, leading to a value of $\kappa_{0}=2.5\pm 0.3$. Such a value of $\kappa_{0}$ is the lowest found in our studies for f=0.95 and reflects the increased correlations, which are induced when ordinary collisions disappear. The effect of controlled collisions to turn the MB distribution of energies to a kappa distribution is reminiscent of the effect of preferential attachment in complex networks [83,84] that turns the degree distribution from exponential to power law in scale free networks, see, e.g., Chapter 5 of [85].

We now turn to the models where the ordinary collision step between the particles of the CP is accompanied by pseudo-collisions of either short range (i.e., between nearest neighbors) or long range (i.e., between non-adjacent particles), e.g., see Figure 5. Such models lead to $\kappa_{0}$ values, in a form compatible with that found by Livadiotis [80], that simulate the effect of an interaction potential, which depends on the parameter that governs the range.

Turning now to models that induce a correlation cluster around the particles that pseudo collide, the results presented in Section 2.3 revealed that multiparticle collisions are not a physically plausible model since they lead to Gaussian like energy distributions. Thus, the formation of the correlated cluster should be based on controlled collisions which are short-range and involve only two particles at each instant (cf. the latter is also compatible with the finite speed of light that controls interactions in nature).

**Figure 17:**
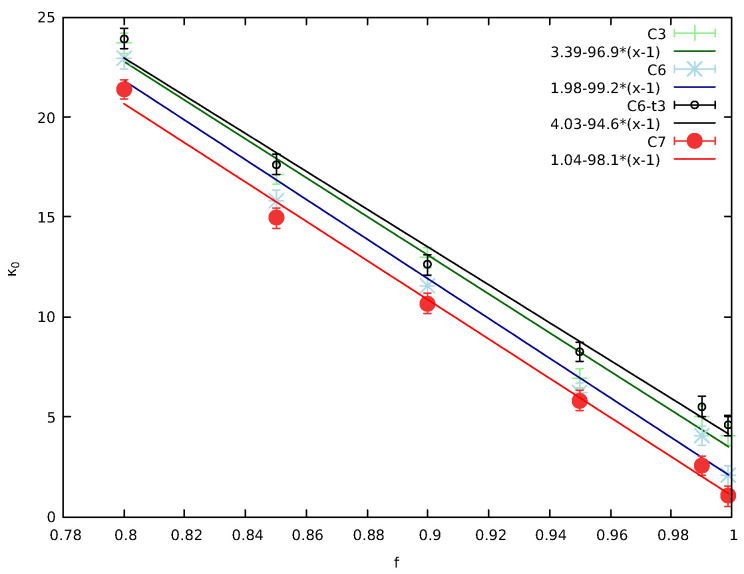
The values of $\kappa_{0}$ obtained from various models of Figure 10 and Figure 12 that include a correlated cluster versus *f*. As we increase the number of correlation time steps, correlation increases and $\kappa_{0}$ approaches 0. The introduction of ordinary collisions frustrates the correlated cluster and effectively cancel the correlation steps.

Thus, we studied in Section 2.4 two classes of models (see Figure 10 and Figure 12) in which the correlation cluster increased its size by correlation time steps in which the neighbors around the original *j* and j+1 continued to pseudo-collide each other. A summary figure for these two classes of models is provided in Figure 17, which shows that such models are able to lower $\kappa_{0}$ as *f* increases to larger and larger values close to its physical limit of 0. Moreover, when including more ordinary collisions within the correlated cluster these effectively “cancel” the correlation time steps increasing $\kappa_{0}$ (see the black line in comparison to the green one in Figure 17) providing a dynamic equilibrium between mechanisms that decrease or increase correlations.

The present study extends our knowledge on how kappa distributions may arise from kinetic models suggested by McComas et al. [18] towards our efforts to provide appropriate simplified models that may describe heliospheric plasmas.

## 4. Conclusions

In summary, in this paper we studied a broad variety of models that are variations of that suggested by McComas et al. [18]. These models are characterized by (i) long-range or short-range patterns, (ii) collision pairs (CP), (iii) multi-particle interactions, and (iv) correlation clusters.

We found that:

1. when long-range correlations are interwoven with CP the resulting thermodynamic kappa are described as an “interatomic” potential interaction among the particles;
2. searching for a closer description of heliospheric plasmas, we found that pairwise correlations of the short-range type are sufficient to lead to the appropriate values of thermodynamic kappa especially when combined with the formation of correlated clusters;
3. simultaneous multi-particle correlation interactions do not lead to thermodynamic stationary states like kappa distributions;
4. the optimal model mimics exactly the stationary states in space plasmas described by kappa distributions, which are characterized by long-range interactions and pair collisions within a cluster of correlations.

Additionally, we observed that when correlations are turned off and collisions persist, the distribution returns to the state of classical thermal equilibrium (Maxwell-Boltzmann distribution) described by independent particles (e.g., see Figure 14). When collisions are turned off, kappa approaches the anti-equilibrium state, e.g., see Figure 16A,C. Finally, in general, the finite kappa is determined by the competing factor of collisions and correlations.

Thus, this paper provides further details on how our simple model can lead to kappa distributions describing space plasmas. Appropriate thermodynamic kappa values can be obtained by mitigating the effect of collisions by correlation clusters and vice versa. In the future, we plan to continue our investigations to microscopically simulate even more complicated concepts of plasmas like the Debye sphere. 

## Data Availability

The original contributions presented in this study are included in the article material. Further inquiries can be directed to the corresponding author.
